# The Efficacy of Simultaneous Injection of Dexamethasone Implant and Ranibizumab Into Vitreous Cavity on Macular Edema Secondary to Central Retinal Vein Occlusion

**DOI:** 10.3389/fphar.2022.842805

**Published:** 2022-03-01

**Authors:** Xing Du, Yanjuan Sheng, Yeqiang Shi, Min Du, Yuanyuan Guo, Shanshan Li

**Affiliations:** Department of Ophthalmology, Jinan Second People’s Hospital, Jinan, China

**Keywords:** dexamethasone implants, vascular endothelial growth factor, retinal vein occlusion, macular edema, intravitreal injection

## Abstract

The purpose of this study was to determine the safety and effectiveness of simultaneous vitreous injection of dexamethasone implant and ranibizumab on macular edema secondary to central retinal vein occlusion (CRVO). We conducted a 6-month retrospective self-control study. Twenty-five patients diagnosed with macular edema secondary to CRVO were enrolled in this study. The patients received intravitreal injection of dexamethasone implant and ranibizumab. The changes in best corrected visual acuity (BCVA), central retinal thickness (CRT) and interocular pressure (IOP) before and at 2w, 1, 2, 3, 4, 5, 6 m after injection were recorded and compared. The adverse reactions in eyes and whole body were observed. The BCVA of all patients at 2 w (61.8 ± 5.42), 1 m (68.68 ± 5.23), 2 m (70.8 ± 5.8), 3 m (68.44 ± 5.61), 4 m (65.76 ± 5.76), 5 m (67.08 ± 5.57), and 6 m (70.12 ± 5.46) after surgery were significantly higher than that before surgery (52.2 ± 5.06,*p* < 0.01), and CRT of all patients at 2w (393.36 ± 52.66 um), 1 m (334.52 ± 32.95 um), 2 m (298.800 ± 29.97 um), 3 m (309.080 ± 28.78 um), 4 m (345.48 ± 39.81 um), 5 m (349.080 ± 29.88 um), and 6 m (309.76 ± 30.41 um) after surgery were significantly reduced than that before surgery (583.76 ± 121.09 um, *p* < 0.01). Macular edema recurred in an average of 4.44 ± 0.51 months after treatment, and those patients received combined treatment again. During follow-up, the most common adverse reactions were subconjunctival hemorrhage and increased intraocular pressure, with the incidence of 22% (11/50) and 18% (9/50) respectively. In all cases, the increased intraocular pressure could be controlled by a single intraocular pressure reducing drug. No patient needed to receive anti-glaucoma surgery. The overall incidence of lens opacity was 4% (2/50). After the first injection, no case showed lens opacity. After re-injection, 2 patients (2 eyes) (8%) developed lens opacity. None of the patients showed serious ocular adverse reactions or systemic complications such as vitreous hemorrhage, retinal detachment, endophthalmitis, uveitis or ocular toxicity. The simultaneous vitreous injection of dexamethasone implant and ranibizumab can significantly improve the visual acuity and anatomical prognosis in macular edema secondary to central retinal vein occlusion (CRVO-ME) patients, exhibiting good safety and effectiveness.

## 1 Introduction

Retinal vein occlusion (RVO) is a retinal vascular disease common in middle-aged and elderly people. The prevalence rate of RVO in people over 40 years old is 1–2% (Rogers et al., 2010), which is only secondary to diabetic retinopathy. The incidence of RVO also increases with age, especially for people over 70 years old (Cugati et al., 2006). In recent years, since the work and lifestyle have changed significantly, the increased psychological pressure and mental stress have led to RVO occurrence in younger population (Laouri et al., 2011). Central retinal vein occlusion (CRVO) is a serious type of RVO that severely impairs the eyesight of patients. The multi-center study involving more than 70,000 people has shown that the prevalence rate of CRVO is 0.8‰, which does not significantly differ between men and women (Rogers et al., 2010). The causes of visual impairment in CRVO patients are complex and diverse, and the main cause is secondary macular edema (ME) (Hayreh et al., 2011; Campochiaro et al., 2013). The current treatment methods for macular edema secondary to central retinal vein occlusion (CRVO-ME) include surgical treatment, laser treatment, intravitreal injection of anti-vascular endothelial growth factor (anti-VEGF) drugs, glucocorticoids and other preparations. Among them, intravitreal injection of anti-VEGF drugs (ranibizumab, Lucentis) is more effective than retinal laser photocoagulation (Kriechbaum et al., 2008; Brown et al., 2011), and it can significantly improve vision and anatomical structure (Heier et al., 2012; Brown et al., 2013). However, clinical observations have found that some patients do not respond well to anti-VEGF drugs, and macular edema still persists under regular anti-VEGF drug treatment (Pulido et al., 2016; Bajor et al., 2017). Studies have shown that inflammatory factors play an important role in the occurrence and development of RVO-ME, and glucocorticoid drugs have an anti-inflammatory effect, which can reduce the level of inflammatory factors, stabilize vascular permeability, and indirectly inhibit VEGF, thus promoting the regression of RVO-ME (Zhao et al., 2011; Rezar-Dreindl et al., 2017). Dexamethasone intravitreal implant (Ozurdex, Allergan) is a biodegradable sustained-release implant loaded with 0.7 mg dexamethasone (Robinson and Whitcup, 2012; Bucolo et al., 2018). Currently, it is the only glucocorticoid drug approved by the US Food and Drug Administration and the European Union for treating RVO-ME. Multi-center clinical trials have shown that intravitreal injection of Ozurdex alone is safe and effective for treating RVO-ME (Georgalas et al., 2019; Hu et al., 2019; Ming et al., 2020). A single intravitreal injection of Ozurdex can maintain the effective drug concentration in the eye for 3–6 months, and it can also be used for RVO patients with poor anti-VEGF treatment efficacy (Manousaridis et al., 2017; Houben et al., 2018; Li et al., 2018). Other drug delivery systems are developing such as nanosystems to deliver drugs to the back of the eye (Amadio et al., 2016). Despite the efficacy of the monotherapy patients can experience recurrence (Pielen et al., 2013) and need multiple and ongoing injections (Horner et al., 2020). Therefore, patients have difficulty in follow-up; in addition national health systems, medical insurances and/or patients themselves also face a certain degree of economic pressure. Lawrence et al. (Iu et al., 2015) found that the reinjection of ranibizumab at 4 weeks after Ozurdex injection was more effective than Ozurdex alone in the treatment of RVO-ME. So we want to try combination therapy to reduce the burden of patients. However, simultaneous injection of Ozurdex and ranibizumab for treating RVO-ME have not been reported. Therefore, in this study, we explored the effectiveness and safety of simultaneous injection of dexamethasone intravitreal implant and ranibizumab in the treatment of CRVO-ME patients.

## 2 Materials and Methods

### 2.1 Patient Selection

Inclusion criteria: (Rogers et al., 2010): Age> 18 years old, patients diagnosed with CRVO by fluorescein fundus angiography (FFA) at first visit, OCT examination showed retina macular thickening and the involvement of fovea (Cugati et al., 2006); The patients completed the follow-up visit at each time point of 2 w, 1, 2, 3, 4, 5, and 6 m after surgery, and the results of BCVA, CRT, and interocular pressure (IOP) were complete (Laouri et al., 2011). During the follow-up period, patients with ME recurrence received the combined treatment of dexamethasone implant and ranibizumab again. Exclusion criteria (Rogers et al., 2010): Patients with history of other eye diseases that affect vision, including diabetic retinopathy, refractive interstitial turbidity, etc. (Cugati et al., 2006); OCT suggested the presence of macular membrane or vitreous macular traction, and vitrectomy was required (Laouri et al., 2011); Patients received intravitreal injection of anti-VEGF drugs or glucocorticoid therapy or had a history of systemic glucocorticoid use (Hayreh et al., 2011); Patients received laser photocoagulation or intraocular surgery within the past 3 months (Campochiaro et al., 2013) Patients with glaucoma, history of elevated intraocular pressure, or shallow anterior chamber (Kriechbaum et al., 2008); Patients with other retinopathy that can cause macular edema (Brown et al., 2011). Patients with other factors that might affect the study results, as assessed by the investigator.

This study was approved by the Ethics Committee of the Jinan Second People’s Hospital and followed the “Declaration of Helsinki” (Registration No. JNEYLL201912006).

### 2.2 Method

#### 2.2.1 General Information

This study was a retrospective case series study, and entailed a review of the medical records of eligible subjects. Fifty-six patients who were diagnosed with ME secondary to CRVO and received simultaneous injection of dexamethasone implant (Ozurdex, Allergan) and ranibizumab (Lucentis, Novartis, Basle, Switzerland) were collected at the Department of Ophthalmology of Jinan Second People’s Hospital from January 2020 to April 2021. Among the 39 patients who received retreatment, there were 25 patients (25 eyes) met the inclusion criteria. Including 11 cases of ischemic type and 14 cases of non-ischemic type. There were 14 male and 11 female patients, with an average age of 54.47 + 7.89 years, ranging from 36 to 70 years old. There were 13 cases with right eye, 12 cases with left eye, and 3 cases with intraocular lens (Table 1). Dexamethasone implant and ranibizumab were simultaneously injected into the vitreous cavity.

**TABLE 1:** Demographic characteristics of the study population.

Number of patients	Age (Years)	Gender (M/F)	Eye (L/R)	IOL (Y/N)	Ischemic type (Y/N)
25	54.47 + 7.89	14/11	12/13	3/22	11/14

M = Man, F=Female. L = Left, R = Right. IOL = intraocular lens. Y=Yes, N=No.

#### 2.2.2 Eye Examination

All patients received a comprehensive eye examination. The best corrected visual acuity (BCVA) examination was performed using the Early Treatment Diabetic Retinopathy Study (ETDRS) eye chart. The non-contact tonometer (NT-510, NIDEK, Japan) was used to measure the intraocular pressure (IOP) of the affected eye. An OCT instrument (Cirrus OCT from Zeiss, Germany) with central retinal thickness (CRT) ≥ 300 μm was used to observe the macular fovea CRT thickness within the range of 1 mm.

#### 2.2.3 Surgery

The intravitreal injection operations were all performed in a sterile laminar flow operating room by the same experienced physician. Lacrimal passage was flushed to rule out dacryocystitis at 3 days before the operation, and levofloxacin eye drops were applied to the operation eye 4 times a day. The pupils were dilated with 0.5% compound tropicamide eye drops at 20 min before injection. Before injection, routine conjunctival sac washing and eye disinfection were performed on the operation eye. Proparacaine hydrochloride eye drops were used for surface anesthesia. After disinfection, the conjunctival sac was rinsed with normal saline. A needle was inserted 3.5 mm behind the superior nasal limbus to inject 0.05 ml (0.5 mg) of Lucentis into the vitreous cavity. The needle was quickly withdrawn and a sterile cotton swab was used to compress the injection site to prevent vitreous incarceration and drug reflux. After 5 min, a 22G special syringe was used to inject 0.7 mg of dexamethasone implant (Ozurdex) into the vitreous cavity at 3.5 mm behind the superior temporal corneal limbus. After the needle was withdrawn, a sterile cotton swab was used to pressurize the injection site to prevent vitreous incarceration. At the same time, the floatation status of the implant was checked. When the intraocular pressure checked by finger palpation was normal, the tobramycin dexamethasone ointment (TobraDex, Alcon) was applied in the conjunctival sac and the operation eye was covered with sterile gauze.

#### 2.2.4 Follow-Up and Observation Indicators

On the first day after the injection, the patient was observed for post-injection reactions, and the BCVA and intraocular pressure were measured. Regular follow-up visits were performed at 2 weeks (2w), 1 month (1 m), 2 months (2 m), 3 months (3 m), 4 months (4 m), 5 months (5 m), and 6 months (6 m) after injection; during the follow-up, BCVA, intraocular pressure, fundus color photography and OCT examination were performed with the same equipment and method as before. If fluorescein fundus angiography (FFA) examination found non-perfusion area in the retina, the laser photocoagulation treatment would be conducted on the peripheral retinal area. During follow-up, if CRT was found to increase by more than 50 um from the lowest recorded level, or visual acuity decreased by 6 letters, the combined injection of vitreous dexamethasone implant and ranibizumab was performed again.

### 2.3 Statistical Analysis

SPSS 23.0 statistical software was used for statistical analysis. The measurement data was expressed as mean ± SD (x±sd) and tested by Shapiro-Wilk to see if the data was normally distributed. Comparison between normally distributed data used One-way repeated measures ANOVA. Non-normally distributed data was tested by paired-sample Wilcoxon rank sum test. At 2 months after operation, the intraocular pressure results of 25 patients were non-normally distributed, the data was expressed in quartile, and the rest of the measurement data were all normally distributed. *p* < 0.05 was considered statistically significant.

## 3 Results

The specific results of BCVA, CRT, and IOP changes are shown in Table 2.

**TABLE 2:** Results of BCVA, CRT, and IOP at each time point before and after surgery (x ± sd).

	BCVA (letter number)	CRT (um)	IOP (mmHg)
pre-op	52.2 ± 5.06	583.76 ± 121.09	15.12 ± 2.24
2w post-op	61.8 ± 5.42	393.36 ± 52.66	16.08 ± 2.21
1 m post-op	68.68 ± 5.23	334.52 ± 32.95	17.92 ± 2.23
2 m post-op	70.8 ± 5.8	298.800 ± 29.97	19 (16, 22.5)
3 m post-op	68.44 ± 5.61	309.080 ± 28.78	17.72 ± 2.07
4 m post-op	65.76 ± 5.76	345.48 ± 39.81	16.12 ± 2.01
5 m post-op	67.08 ± 5.57	349.080 ± 29.88	16.44 ± 1.61
6 m post-op	70.12 ± 5.46	309.76 ± 30.41	18 ± 4.42

### 3.1 BCVA

**FIGURE 1:**
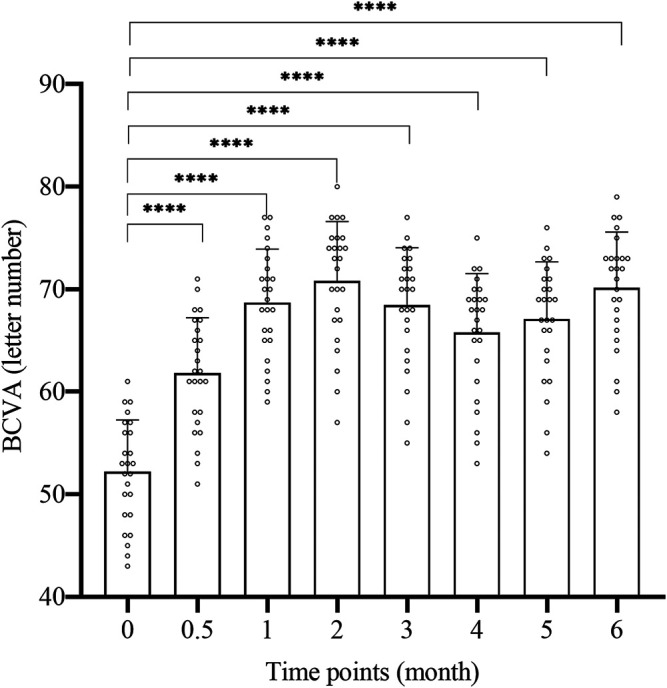
The BCVA change of each time. BCVA: best corrected visual acuity. 0: pre-op, 0.5: 2w post-op, 1: 1 m post-op, 2: 2 m post-op, 3: 3 m post-op, 4: 4 m post-op, 5: 5 m post-op, 6: 6 m post-op. ****: Comparison between two time points *p* < 0.0001.

The BCVA change of 25 patients at each time point of 2w, 1, 2, 3, 4, 5, and 6 m after surgery is shown in Figure 1 and Table 2. The letter number of BCVA at each time point after surgery was significantly increased compared to before surgery (52.2 ± 5.06), and the difference was significant (*p* < 0.01, Figure 1). The peak of treatment efficacy appeared at 2 m after surgery (70.8 ± 5.8). After reinjection, it increased again at 6 months after surgery (70.12 ± 5.46). Moreover, the BCVA of both non-ischemic CRVO and ischemic CRVO were increased at each time point after surgery compared with before surgery, and the difference was significant (*p* < 0.01).

### 3.2 CRT

**FIGURE 2:**
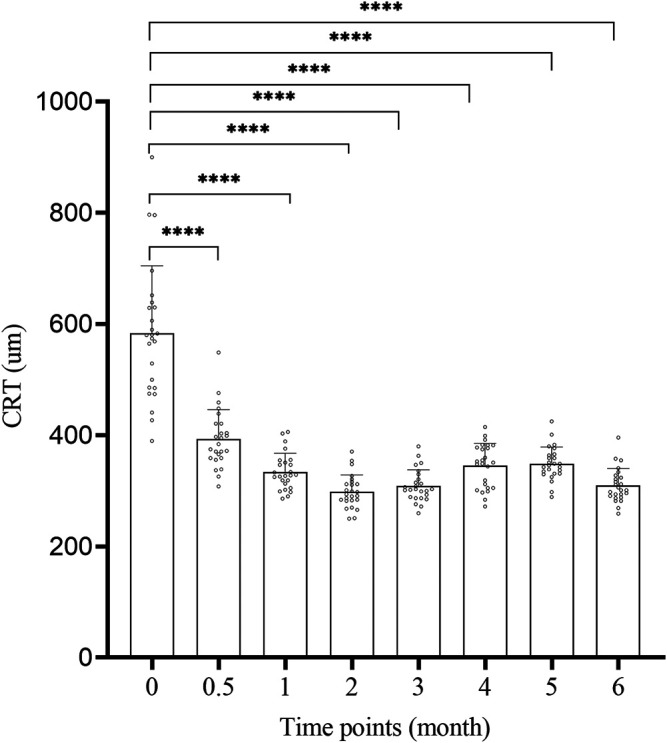
The CRT change of each time. CRT: central retinal thickness. 0: pre-op, 0.5: 2w post-op, 1: 1 m post-op, 2: 2 m post-op, 3: 3 m post-op, 4: 4 m post-op, 5: 5 m post-op, 6: 6 m post-op. ****: Comparison between two time points *p* < 0.0001.

The CRT change of 25 patients at 2w, 1, 2, 3, 4, 5, 6 m after surgery is shown in Figure 2 and Table 2. The CRT at each time point after surgery was significantly lower than that before surgery (583.76 ± 121.09um), and the difference was significant (*p* < 0.01, Figure 2). The peak of treatment efficacy occurred at 2 m after surgery (298.800 ± 29.97 um). After reinjection, it decreased again at 6 months after surgery (309.76 ± 30.41 um). Moreover, the CRT of both non-ischemic CRVO and ischemic CRVO at each time point after surgery was lower than that before surgery, and the difference was significant (*p* < 0.01).

### 3.3 Recurrence of Macular Edema

Among these patients, macular edema (ME) recurred in an average of 4.44 ± 0.51 months after treatment. The patients with ME recurrence received a second combined injection of dexamethasone implant and ranibizumab.

### 3.4 Adverse Reactions

In this study, 11 cases showed subconjunctival hemorrhage at the injection site (10 eyes), overall incidence was 22% (11/50), of which 5 cases showed subconjunctival hemorrhage after the first injection (5 eyes, accounting for 20%), and 6 cases showed subconjunctival hemorrhage after re-injection (6 eyes, accounting for 24%). All subconjunctival bleeding could be absorbed by itself without treatment.

The IOP change of 25 patients at 2 w, 1, 2, 3, 4, 5, 6 m after surgery is shown in Table 2.

A total of 9 cases (7 eyes) had increased postoperative intraocular pressure after treatment, overall incidence was 18% (9/50), of which 6 cases had an intraocular pressure ≥21 mmHg after the first injection, accounting for 24% (6/25), and 4 cases had an increase of ≥10 mmHg compared to before injection, accounting for 16% (4/25). After re-injection, 3 patients had an intraocular pressure ≥21 mmHg, accounting for 12% (3/25); 1 patient had an increase of ≥10 mmHg, accounting for 4% (1/25). In all cases, a single intraocular pressure-reducing drug was enough to control intraocular pressure, and no patients showed secondary glaucoma or needed surgical treatment. The overall incidence of lens opacity was 4% (2/50). After the first injection, no case showed lens opacity. After re-injection, 2 patients (2 eyes) (8%) developed lens opacity, which was subcapsular opacity, and no surgical treatment was required. In addition, no patient showed severe ocular adverse reactions such as vitreous hemorrhage, retinal detachment, endophthalmitis, uveitis, or ocular toxicity, and no systemic complications such as cardiovascular and cerebrovascular diseases were observed (Table 3).

**TABLE 3:** Adverse events of this study.

	—	First injection	Re-injection	Total
Subconjunctival hemorrhage	—	5/25 (20%)	6/25 (24%)	11/50 (22%)
Intraocular pressure increase	≥21 mmHg	6/25 (24%)	3/25 (12%)	9/50 (18%)
—	Increase≥10 mmHg	4/25 (16%)	1/25 (4%)	5/50 (10%)
Cataract	—	0	2/25 (8%)	2/50 (4%)

## 4 Discussion

The pathogenesis of RVO-ME is a complex process, in which inflammatory factors and vascular endothelial growth factor play an important role (Campochiaro et al., 2008). At present, intravitreal anti-VEGF agents and intravitreal corticosteroid agents are the effective therapies for CRVO-ME (Singer et al., 2012; Qian et al., 2018). American Academy Of Ophthalmology and EURETINA clarified that anti-VEGF drugs such as ranibizumab and aflibercept are the first-line treatment for RVO-ME, while panretinal laser photocoagulation (PRP) and glucocorticoid are the second-line treatment for RVO-ME (Pulido et al., 2016; Schmidt-Erfurth et al., 2019). By examining the aqueous humor and vitreous body, Noma et al. found that the VEGF level was increased in RVO patients, and the concentration of VEGF was positively correlated with the degree of macular edema (Noma et al., 2006). Therefore, anti-VEGF therapy has become the main treatment for RVO-ME. Inflammatory factors such as angiotensin II and prostaglandins are also involved in the pathogenesis of RVO-ME (Manousaridis et al., 2017). The interaction between inflammation and vascular pathological changes can aggravate the disease progression in RVO patients (Yoshimura et al., 2009). In addition, inflammatory response and/or ischemia may lead to further increase in VEGF levels and aggravate the progression of macular edema. Glucocorticoids are a family of drugs that can inhibit inflammatory factors, reduce retinal exudation, and stabilize the blood-retinal barrier (Floman and Zor, 1977). At the same time, glucocorticoids can inhibit VEGF pathway (Fischer et al., 2001), inhibit retinal vascular leakage, decrease VEGF expression (Edelman et al., 2005; Felinski and Antonetti, 2005; Bucolo et al., 2018), reduce blood vessel exudation, thus reducing macular edema. Dexamethasone intravitreal implant is an effective glucocorticoid medicine with anti-inflammatory activity, and it can be used to treat macular edema complicated by vascular retinal diseases including RVO. Studies have shown that the dexamethasone implants in vitreous cavity can reduce the levels of pro-inflammatory cytokines MCP-1, IL17-E, IL-1a in RVO, and the decrease of these cytokines is related to the decrease of retinal thickness in the central area of the macula (Rezar-Dreindl et al., 2017). Therefore, anti-inflammatory and anti-VEGF treatments can be combined in the clinical treatment of RVO-ME, especially for the severe type, CRVO. Thus, in this study, we tried to inject dexamethasone and anti-VEGF drugs into the vitreous cavity at the same time to verify its effectiveness and safety.

### 4.1 Effectiveness

We compared the improvements of BCVA and macular edema after treatment with the results before surgery, and the difference was obvious. Compared with the preoperative level (52.2 ± 5.06 letters), the average BCVA of the treated eye was already greatly improved at 2 weeks after injection, and was significantly better than pre-operative level at all time points after injection. The BCVA increased most significantly at 2 months after surgery, with an average increase of 18.6 letters compared to the preoperative period (Figure 1; Table 2). These results demonstrate that the simultaneous injection of dexamethasone implant and ranibizumab into intravitreal cavity can significantly improve the BCVA in CRVO patients. The average preoperative CRT was 583.76 ± 121.09 um. There was a significant decrease in CRT at 2 weeks after surgery, and the CRT at all time points was significantly lower than preoperative level. The CRT decreased most significantly at 2 months after surgery, with an average of 298.800 ± 29.97, which was reduced by 284.96 um compared with the preoperative level (Figure 2; Table 2). These results confirm that the intravitreal injection of dexamethasone implant combined with ranibizumab can significantly alleviate the degree of macular edema in CRVO patients. In this study, the average recurrence time of macular edema was 4.44 ± 0.51 months, which was longer than the recurrence time from dexamethasone implant treatment alone (Li et al., 2018), indicating that the combination therapy has better durability than single treatment.

The above results suggest that the reason why the patients had better functional and anatomical improvements after surgery might be the synergistic effect from different mechanisms of dexamethasone intravitreal implants (anti-inflammatory drugs) and ranibizumab (anti-VEGF drugs), which resulted in faster, more pronounced and longer BCVA growth and improvement in macular edema. Previous studies have shown that the greatest effect of dexamethasone intravitreal implants (the peak effect) occurs around 2 months after injection (Haller et al., 2010; Li et al., 2018), which is consistent with our results. Therefore, our results demonstrate that the combined injection (dexamethasone implant and ranibizumab) integrates the advantages of the two treatments, and the effect is more significant.

### 4.2 Safety

The common adverse events in this study were subconjunctival hemorrhage and glucocorticoid-related intraocular pressure increase, which were consistent with the findings from Li, Haller, et al. (Haller et al., 2010; Haller et al., 2011; Li et al., 2018). The cases with subconjunctival hemorrhage did not require treatment and the hemorrhage could be absorbed by itself. The incidence of subconjunctival hemorrhage can also be reduced by avoiding conjunctival blood vessels during injection. Previous studies have shown that the incidence of secondary glaucoma after intravitreal injection of glucocorticoids is 7.0–28.5% (Malclès et al., 2017; Li et al., 2018). This rate is closely related to time point, statistical method, and sample size. In this study, the intraocular pressure increased in 24% of patients after the first injection, and the increase in intraocular pressure was most obvious at 2 months after injection, which was consistent with previous results (Haller et al., 2010; Li et al., 2018). After the second injection, the intraocular pressure increased in 16% of patients, which might be due to the short observation time after re-injection. In this study, all cases with elevated intraocular pressure could be controlled by a local intraocular pressure-reducing drug, and no patients needed to undergo anti-glaucoma surgery. We found that postoperative intraocular pressure increased most significantly at 2 months after surgery. Combined with previous reports, we suggest that special attention should be paid to the monitoring of intraocular pressure within 3 months after injection; especially during the first 2 months with high levels of glucocorticoids and major treatment effect, it is better to monitor intraocular pressure weekly. If the intraocular pressure after injection is increased by ≥10 mmHg or ≥25 mmHg, the patient needs to take intraocular pressure-reducing drug. Cataract is one of the main side effects of glucocorticoid therapy, and its progression is an important research focus. So far, it is generally believed that the proportion of cataract progression during the 6-month follow-up is not high, ranging from 7.3 to 13.3% (Haller et al., 2010; Coscas et al., 2014). Previous studies on RVO treatment with dexamethasone have shown that, when multiple intravitreal injections of dexamethasone are applied and treatment time is long, cataract progression is likely to occur and the patients may need cataract surgery (Haller et al., 2011; Reid et al., 2015). In this study, no aggravation of lens opacity was found after the first injection. During 6 months of follow-up, 2 cases (2 eyes) showed worsening of lens opacity, which was more obvious in the posterior capsule. Both patients were re-injection patients, and surgical treatment of cataract was not needed, which is similar to the results from Haller and Reid (Haller et al., 2011; Reid et al., 2015).

The results of this study showed that the simultaneous injection of dexamethasone implant (Ozurdex) and ranibizumab (Lucentis) into the vitreous cavity could significantly improve the visual acuity and anatomical prognosis of CRVO-ME patients. Moreover, no serious complications occurred in these patients. Therefore, the combined application of anti-VEGF and anti-inflammatory drugs played a complementary and synergistic role, showing good effectiveness and safety.

This study is a single-center study, which has some limitations such as small sample size and short follow-up time. Moreover, the patients with branch retinal vein occlusion were not included. In the future, a multi-center, prospective, and randomized controlled study with large sample size is needed to provide more evidence.

## Data Availability

The original contributions presented in the study are included in the article/Supplementary Material, further inquiries can be directed to the corresponding author.
